# Recent illicit drug use among psychiatric patients in Brazil: a national representative study

**DOI:** 10.11606/S1518-8787.2017051006543

**Published:** 2017-08-03

**Authors:** Miriam Almeida Nahas, Ana Paula Souto Melo, Francine Cournos, Karen Mckinnon, Milton Wainberg, Mark Drew Crosland Guimarães

**Affiliations:** I Programa de Pós-Graduação em Saúde Pública. Faculdade de Medicina. Universidade Federal de Minas Gerais. Belo Horizonte MG, Brasil; II Faculdade de Medicina. Universidade Federal de São João del-Rei. Divinópolis, MG, Brasil; III Mailman School of Public Health. Columbia University. New York, USA; IV New York State Psychiatric Institute. Columbia University. New York, USA; V New York State Psychiatric Institute. Columbia University. New York, USA; VI Departamento de Medicina Preventiva e Social. Faculdade de Medicina. Universidade Federal de Minas Gerais. Belo Horizonte, MG, Brasil

**Keywords:** Mentally Ill Persons, Street Drugs, Risk Factors, Gender and Health, Multicenter Study

## Abstract

**OBJECTIVE:**

To estimate factors associated to illicit drug use among patients with mental illness in Brazil according to gender.

**METHODS:**

A cross-sectional representative sample of psychiatric patients (2,475 individuals) was randomly selected from 11 hospitals and 15 public mental health outpatient clinics. Data on self-reported illicit drug use and sociodemographic, clinical and behavioral characteristics were obtained from face-to-face interviews. Logistic regression was used to estimate associations with recent illicit drug use.

**RESULTS:**

The prevalence of any recent illicit drug use was 11.4%. Men had higher prevalence than women for all substances (17.5% and 5.6%, respectively). Lower education, history of physical violence, and history of homelessness were associated with drug use among men only; not professing a religion was associated with drug use in women only. For both men and women, younger age, current hospitalization, alcohol and tobacco use, history of incarceration, younger age at sexual debut, and more than one sexual partner were statistically associated with illicit drug use.

**CONCLUSIONS:**

Recent illicit drug use among psychiatric patients is higher than among the general Brazilian population and it is associated with multiple factors including markers of psychiatric severity. Our data indicate the need for the development of gender-based drug-use interventions among psychiatric patients in Brazil. Integration of substance use treatment strategies with mental health treatment should be a priority.

## INTRODUCTION

Illicit drug abuse and dependence are important public health issues worldwide. Data from the World Health Organization (WHO) show that substance use and mental disorders are, together, the number one cause of years lost due to disability (22% of YLD) and the 15 highest cause of disability-adjusted life years (7.4% of DALYs)^32^. During 2010, 3.4% to 6.6% of the worldwide adult population used some illicit substance, and 10% to 13% of this population demonstrated abuse or dependence. Illicit drug use greatly increases the probability of having health risk behavior and is associated with higher rates of overdose, suicide, violence, HIV infection, and sexual risk behavior^26,30^. Injecting drugs further increases these risks, and needle sharing may transmit blood-borne diseases among users^30^.

The association between mental disorders and psychoactive substance use has been frequently discussed in the literature since the 1990s. Despite varying prevalence rates in different countries^22^, high prevalence of illicit drug use in psychiatric patients is evident^2,14,18,23^. Data from the USA indicate that, while 3.7% of the persons without history of mental illness had a history of lifetime drug abuse or dependence, the rate was 14.7% (OR = 4.7; 95%CI 3.5–6.3) among those with any lifetime mental illness diagnosis^23^. In the United Kingdom, Fisher et al.^6^ identified that psychiatric patients had twice the chance of developing a substance use disorder as compared to those with no mental disorders. A study carried out in São Paulo, Brazil, showed that 4.7% of adult patients with severe mental illness (SMI) in treatment fulfilled criteria for abuse or dependence of illicit drug use and 8.3% had used an illicit substance in the past year^19^. A current comorbid substance use disorder was present in 11.2% of a sample of outpatients with SMI in Rio de Janeiro, Brazil^31^.

Illicit drug use is associated with multiple factors, including child sexual abuse, parental violence, parents or sibling who also use drugs, and being younger, male, single, and with lower schooling^3,5^. Similar to the general population, drug use among patients with mental illness is higher among those who are male, younger^3,11,12,18^, single^18^, have family members who use drugs^3^, or have a history of homelessness or incarceration^23^.

There is a remarkable difference in rates of drug use between men and women. Use among women is lower than among men, with differences that vary from a 3:4 ratio in the United States to 1:10 in India and Indonesia^30^, indicating higher vulnerability among men. The only instance in which this ratio is reversed is in the use of tranquilizers and sedatives. Comorbidity between severe mental illness and substance use disorders is more prevalent among men, whereas women with alcohol or drug-associated disorders had twice the risk of having depressive and anxiety disorders when compared to men^29^.

Illicit drug use and mental illness can have a negative impact on health such as reduced adherence to treatment and worsen prognosis, leading to more intensive and expensive treatments in emergency settings^2,6^. However, few surveys on the association between illicit drug use and mental illness have been conducted in low and middle-income countries^11^. Brazil has scarce published data on illicit drug use among patients with mental illness and no studies with a national representative sample. This study aimed to estimate the risk behavior factors associated with recent illicit drug use among patients with mental illness in Brazil according to gender.

## METHODS

We obtained data from a large national multicenter cross-sectional study conducted in 2006. The main objective was to determine the prevalence of infection with HIV, syphilis, and hepatitis B and C in a sample of adult psychiatric patients (18 years of age or older) in treatment in Brazil^20^.

A two-stage sampling procedure was carried out with a random selection of centers in each region followed by random selection of participants within each center^20^. Sample size calculation considered 50% average estimate of the conditions, 0.2% precision, 5% confidence level, and a potential 40% loss. It was proportional to the type of care (hospital or Psychosocial Care Centers [CAPS]) and to the distribution of AIDS cases in the regions. Among the 3,255 patients recruited, 2,763 (84.9%) were considered eligible as previously described^8^. From these patients, 288 (10.4%) did not participate for various reasons (such as refusal, non-attendance, death), amounting to a 26% loss for the interview, below the initial estimate of 40%. Public outpatient mental health clinics that exclusively treated substance use disorders (CAPS AD) were excluded from the sampling process to prevent the overestimation of the selected risk behaviors or prevalence rates. The study was carried out with 2,475 patients recruited from 11 psychiatric hospitals and 15 public mental health outpatient clinics (CAPS) distributed in the five Brazilian macroregions.

Patients who agreed to participate and were considered eligible underwent a face-to-face interview with a semi-structured questionnaire, tested in a preliminary pilot study^8^, to collect information about health care, behavioral characteristics, and sociodemographic profile. Healthcare professionals who worked at the treatment services administered the interviews. Additional clinical data such as psychiatric diagnosis and treatment characteristics were collected from the medical records. The study included only patients able to provide written informed consent and who were able to answer the questionnaire after the administration of a preliminary assessment adapted from the Mini-Mental State Examination (MMSE). Further details have been previously published^8^.

The outcome of interest for this analysis was recent illicit drug use (i.e., patients that reported using any illicit drug at least once during the 12 months prior to the interview). The illicit drugs considered were marijuana, cocaine, crack, hallucinogens, amphetamines, opiates, solvents, or other non-prescribed or illicit drugs.

Potential explanatory variables were divided into three groups: sociodemographic, clinical characteristics, and risk behavior. Sociodemographic characteristics included age, skin color, marital status, schooling, having an income in the last six months, place of residence, and professing a religion. Median age (40 years old) was used as a cutoff point. Skin color was dichotomized as non-white and white. For marital status, those who were single, separated/divorced, or widowed were grouped into one category. The cutoff point for schooling was five years (first cycle of elementary school). For housing, those living in houses or apartments were considered stable, while those living in shelters, pensions, hostels, and on streets were grouped as unstable. Clinical characteristics included main psychiatric diagnosis, history of hospitalization, medical comorbidities, and history of sexually transmitted infections (STI). The main psychiatric diagnosis was classified according to the International Classification of Diseases (ICD-10), defined by a psychiatrist as registered in the medical chart. This variable was dichotomized into severe mental illness (SMI) and compared to the other diagnoses. Severe mental illnesses included diagnoses of schizophrenia, bipolar disorders, and depression with psychotic symptoms^25^. History of hospitalization was analyzed by dividing the sample into three categories: those who were hospitalized in one of the participating psychiatric hospitals during the study; those who were under care in CAPS but had a history of any previous hospitalization; and those who were under care in CAPS and had never been hospitalized. Risk behavior characteristics included the following variables: lifetime tobacco use, lifetime alcohol use, history of homelessness, history of physical and sexual violence, history of incarceration, age of first sexual intercourse, number of partners, and practice of unprotected sex. Lifetime risk behavior self-reports have demonstrated reliability^8^. The cutoff point for age at first sexual intercourse was 18 years. Finally, those with two or more lifetime sexual partners were compared to those with only one or no partners, and unprotected sex was defined as not always using condoms in all sexual practices (ever).

Analyses were conducted separately for men and women. Descriptive analyses were carried out and the prevalence of recent illicit drug use was calculated by dividing the number of participants who reported using any illicit drug in the 12 months prior to the interview by the total number of participants, with 95% confidence interval (95%CI). For the univariate analysis, differences in proportion were assessed by Pearson’s Chi-square test, and the magnitude of the associations was estimated by the odds ratio (OR) with 95%CI. A multivariate logistic model was used to estimate the independent effect of potential explanatory variables, using a sequential backward elimination method. Variables that presented p < 0.20 in the univariate analysis were initially included. Wald test was used to assess the statistical significance of each variable. Only those with p < 0.05 remained in the final model. Goodness of fit was assessed by the Hosmer-Lemeshow test. For statistical analysis, EpiInfo 7 and Stata 12 were used.

## RESULTS

All participants who answered to the questionnaire were included in the study (n = 2,475): 51.6% were women (n = 1,277) and 48.4% were men (n = 1,198). As shown in Table 1, most men and women were 40 years old or over, single, with no income in the last six months, had stable housing, and professed a religion. Men were more likely than women to be single (75.8%) and to live in unstable housing (17.5%), whereas women were more likely than men to profess a religion (87.8%). Men were also more likely than women to have a severe mental illness diagnosis (61.0%), to be hospitalized during the study period (45.0%), and to have a prior history of hospitalization (34.4%). Overall, high prevalence of self-reported medical comorbidities (45.3%) and history of STI (23.3%) was also found. In addition, men had a higher proportion of tobacco smoking, alcohol drinking, early sexual debut (< 18 years old), more than one lifetime sexual partner, and history of incarceration and homelessness, whereas women had a higher proportion of history of sexual violence and lifetime unprotected sex (Table 1).

**Table 1:** Sociodemographic, clinical, and risk behavior characteristics by gender. PESSOAS Project, 2006–2007, Brazil. (n = 2,475)

Characteristic	Men		Women		Total	
		
	(n = 1,198)		(n = 1,277)		(n = 2,475)	
		
	n	%*	n	%*	n	%*
Sociodemographic						
Age (years old)						
< 40	594	49.6	530	41.5	1,124	45.4
≥ 40	604	50.4	747	58.5	1,351	54.6
Skin color						
Non-white	613	51.2	587	46.0	1,200	48.5
White	584	48.8	689	54.0	1,273	51.5
Marital status						
Single/Separated/Divorced/Widowed	907	75.8	746	58.4	1,653	66.8
Married/Common-law marriage/Other	290	24.2	531	41.6	927	33.2
Schooling (years)						
< 5	593	49.5	660	51.7	1,253	50.6
≥ 5	605	50.5	617	48.3	1,222	49.4
Income in last 6 months						
Yes	438	40.6	449	35.2	932	37.8
No	708	59.4	825	64.8	1,533	62.2
Housing						
Unstable	209	17.5	109	8.6	318	12.9
Stable	987	82.5	1,166	91.5	2,153	87.1
Profess any religion						
Yes	935	78.7	1,118	87.8	2,053	83.4
No	253	21.3	155	12.2	408	16.6
Clinical						
Main psychiatric diagnosis						
SMI	731	61.0	682	53.4	1,413	57.1
Non-SMI	467	39.0	595	46.6	1,062	42.9
Psychiatric hospitalization						
Current	537	44.9	361	28.3	898	36.4
Prior	411	34.4	378	29.7	789	31.9
Never admitted	247	20.7	535	42.0	782	31.7
Self-reported medical comorbidity						
Yes	483	40.8	626	49.4	1,109	45.3
No	700	59.2	641	50.6	1,341	54.7
History of STI						
Yes	311	26.4	257	20.4	568	23.3
No	869	73.6	1,003	79.6	1,872	76.7
Risk behavior						
Tobacco use (ever)						
Yes	970	81.3	798	62.9	1,768	71.8
No	223	18.7	470	37.1	693	28.2
Alcohol use (ever)						
Yes	924	77.5	667	52.7	1,591	64.7
No	269	22.5	598	47.3	867	35.3
History of homelessness						
Yes	255	21.5	189	15.0	444	18.1
No	934	78.5	1,071	85.0	2,005	81.9
Physical violence						
Yes	695	58.2	736	57.9	1,431	58.0
No	499	41.8	536	42.1	1,035	42.0
Sexual violence						
Yes	150	12.6	339	26.8	489	19.9
No	1,040	87.4	928	73.2	1,968	80.1
History of incarceration						
Yes	493	41.3	135	10.6	628	25.5
No	701	58.7	1,135	89.4	1,836	74.5
Age of first sexual intercourse						
< 18 years old	632	65	561	50.6	1,193	57.3
≥ 18 years old	341	35	548	49.4	889	42.7
Number of sexual partners						
One or never had sex	292	26	513	42.6	805	34.6
Two or more	832	74	691	57.4	1,523	65.4
Unprotected sex						
Yes	909	76.8	1,055	83.5	1,964	80.2
No	275	23.2	209	16.5	484	19.8

SMI: Severe mental illness

* Total values vary because of missing observations.

The overall prevalence of any illicit drug use was 11.4% in the past year and 25.4% during lifetime (Table 2). Compared to women, men had a higher prevalence of drug use, 17.5% *versus* 5.6% in the past year and 36.8% *versus* 14.7% for lifetime, respectively. For both men and women, the substance used with the highest prevalence was marijuana (8.8% in the past year and 21.9% for lifetime), followed by cocaine (3.4%, in the past year and 10.6% for lifetime). Both were more prevalent among men than women. A small proportion of the sample (2.9%) reported lifetime injection drug use, and use of more than one drug was reported by 24.1% of the men and 5.7% of the women. Finally, men had a higher proportion of drug use during sex (13.4%) and exchange of money or drugs for sex (40.4%) compared to women (5.6% and 13.2%, respectively) (Table 2).

**Table 2:** Descriptive characteristics of illicit drug use (recent and lifetime) by gender. PESSOAS Project, 2006–2007, Brazil. (n = 2,475)

Characteristic	Men		Women		Total	
		
	(n = 1,198)		(n = 1,277)		(n = 2,475)	
		
	n	%*	n	%*	n	%*
Use in the past year						
Marijuana	170	14.2	47	3.7	217	8.8
Cocaine	67	5.6	18	1.4	85	3.4
Solvents	47	3.9	12	0.9	59	2.4
Crack	105	8.8	28	2.2	133	5.4
Hallucinogens	18	1.5	3	0.2	21	0.9
Amphetamine	21	1.8	19	1.5	40	1.6
Opiates	7	0.6	2	0.2	9	0.4
Any illicit drug	210	17.5	71	5.6	281	11.4
Lifetime use						
Marijuana	403	33.6	138	10.8	541	21.9
Cocaine	205	17.1	57	4.5	262	10.6
Solvents	198	16.5	46	3.6	244	9.9
Crack	164	13.7	48	3.8	212	8.6
Hallucinogens	86	7.2	20	1.6	106	4.3
Amphetamine	60	5.0	44	3.5	104	4.2
Opiates	22	1.8	7	0.6	29	1.2
Any illicit drug	441	36.8	188	14.7	629	25.4
Drug use (lifetime)						
None	752	64.1	1,083	85.6	1,835	75.2
One drug only	138	11.8	111	8.8	249	10.2
Two or more drugs	283	24.1	72	5.7	355	14.6
Injection drug use (lifetime)						
Yes	53	4.5	19	1.5	72	2.9
No	1,134	95.5	1,246	98.5	2,380	97.1
Drug use during sexual practices						
Yes	132	13.4	60	5.6	192	9.3
No	853	86.6	1,017	94.4	1,870	90.7
Exchange money/drugs for sex						
Yes	484	40.4	168	13.2	652	26.3
No	714	59.6	1,109	86.8	1,823	73.7

* Total values vary because of missing observations.

In the univariate analysis, most of the variables investigated were statistically associated (p < 0.05) with recent illicit drug use (Table 3) for both men and women, including being younger, having less schooling, previous history of hospitalization, history of STI, and all risk behavior characteristics. On the other hand, being single and having an unstable place of residence were associated with illicit drug use among men only, and not professing a religion and medical comorbidities were associated with women only.

**Table 3:** Univariate analysis of recent illicit drug use stratified by gender. PESSOAS Project, 2006–2007, Brazil. (n = 2,475)

Characteristic	Total*	Men (n = 1,198)					Total*	Women (n = 1 ,277)				
	
		Drug use		OR	95%CI	p		Drug use		OR	95%CI	p
	
		n	%					n	%			
Sociodemographic												
Age (years old)												
< 40	594	168	28.3	5.28	3.68–7.57	0.000	530	45	8.5	2.57	1.56–4.23	0.000
≥ 40	604	42	6.9	1.00			747	26	3.5	1.00		
Skin color												
Non-white	613	108	17.6	1.01	0.75–1.36	0.945	587	38	6.5	1.37	0.85–2.22	0.190
White	584	102	17.5	1.00			689	33	4.8	1.00		
Marital status												
Single/Separated/Divorced/Widowed	907	181	20.0	2.24	1.48–3.40	0.000	746	47	6.3	1.42	0.86–2.35	0.171
Married/Common-law marriage/Other	290	29	10.0	1.00			531	24	4.5	1.00		
Schooling (years)												
< 5	593	146	24.6	2.76	2.00–3.80	0.000	660	45	6.8	1.66	1.01–2.73	0.042
≥ 5	605	64	10.6	1.00			617	26	4.2	1.00		
Income in last 6 months												
Yes	483	85	17.6	0.99	0.74–1.35	0.979	449	28	6.2	1.21	0.74–1.98	0.446
No	708	125	17.7	1.00			825	43	5.2			
Housing												
Unstable	209	24	11.5	0.56	0.35–0.88	0.011	109	6	5.5	1.00	0.42–2.38	0.994
Stable	987	186	18.8	1.00			1,166	64	5.5	1.00		
Profess any religion												
Yes	935	167	17.9	1.00			1,118	55	4.9	1.00		
No	253	43	17.0	0.94	0.65–1.36	0.749	155	16	10.3	2.22	1.24–3.99	0.006
Clinical												
Main psychiatric diagnosis												
SMI	731	125	17.1	0.93	0.68–1.26	0.641	682	41	6.0	1.20	0.74–1.96	0.450
Non-SMI	467	85	18.2	1.00			595	30	5.0	1.00		
Psychiatric hospitalization												
Current	537	145	27.0	3.98	2.45–6.47	0.000	361	41	11.3	6.10	3.09–12.05	0.000
Prior	411	44	10.7	1.29	0.75–2.23	0.360	378	19	5.0	2.52	1.19–5.36	0.016
Never admitted	247	21	8.5	1.00			535	11	2.1	1.00		
Self-reported medical comorbidity												
Yes	483	89	18.4	1.09	0.80–1.48	0.587	626	27	4.3	0.61	0.37–1.00	0.048
No	700	120	17.4	1.00			641	44	6.9	1.00		
History of STI												
Yes	311	76	24.4	1.77	1.29–2.44	0.000	257	23	8.9	1.96	1.17–3.28	0.009
No	869	134	15.4	1.00			1,003	48	4.8	1.00		
Risk behavior												
Tobacco use (ever)												
Yes	970	210	17.6	6.22	3.13–12.33	0.000	798	63	7.9	4.95	2.35–10.42	0.000
No	223	9	4.0	1.00			470	8	1.7	1.00		
Alcohol use (ever)												
Yes	924	197	21.3	5.34	2.99–9.52	0.000	667	58	8.7	4.29	2.32–7.90	0.000
No	269	13	4.83	1.00			598	13	2.2	1.00		
History of homelessness												
Yes	255	78	30.6	2.72	1.97–3.77	0.000	189	24	12.7	3.17	1.89–5.32	0.000
No	934	130	13.9	1.00			1,071	47	4.4	1.00		
Physical violence												
Yes	695	166	23.9	3.33	2.33–4.76	0.000	736	56	7.6	3.07	1.69–5.58	0.000
No	499	43	8.6	1.00			536	14	2.6	1.00		
Sexual violence												
Yes	150	38	25.3	1.74	1.16–2.59	0.000	339	34	10.0	2.9	1.79–4.80	0.000
No	1,040	170	16.4	1.00			928	34	3.7	1.00		
History of incarceration												
Yes	493	136	27.6	3.33	2.43–4.55	0.000	135	23	17.0	4.75	2.78–8.12	0.000
No	701	72	10.3	1.00			1,135	47	4.1	1.00		
Age of first sexual intercourse												
< 18 years old	632	156	24.7	2.27	1.57–3.28	0.000	561	55	9.8	4.15	2.28–7.55	0.000
≥ 18 years old	341	43	12.6	1.00			548	14	2.6			
Number of sexual partners												
Only one or never had sex	292	12	4.1	1.00			513	5	1.0	1.00		
Two or more	832	188	22.6	6.81	3.73–12.41	0.000	691	63	9.1	10.19	4.07–25.53	0.000
Unprotected sex												
Yes	909	183	20.1	2.63	1.68–4.13	0.000	1,055	66	6.3	4.58	1.43–14.72	0.005
No	275	24	8.7	1.00			209	3	1.4	1.00		

SMI: Severe mental illness

* Total values vary because of missing observations.

The final multivariate models (Table 4) indicated that age (< 40 years old), history of hospitalization, lifetime tobacco use, lifetime alcohol use, history of incarceration, age at sexual debut (< 18 years old), and number of sexual partners (two or more) remained independently associated (p < 0.05) with illicit drug use for both genders. In addition, not professing a religion also remained statistically significant among women, and lower schooling (< 5 years), history of homelessness, and history of physical violence remained statistically significant among men in the respective final models. Having an SMI diagnosis did not remain significant.

**Table 4:** Multivariate analysis stratified by gender. PESSOAS Project, 2006–2007, Brazil. (n = 2,358)

Characteristic	Men (n = 1,146)			Women (n = 1,212)		
	
	OR	95%CI	p	OR	95%CI	p
Sociodemographic						
Age (years old)						
< 40	6.12	4.06–9.26	0.000	1.87	1.06–3.30	0.030
≥ 40	1.00			1.00		
Schooling (years)						
< 5	2.72	1.84–4.02	0.000	-	-	-
≥ 5	1.00			-	-	-
Profess a religion						
Yes	-	-	-	1.00		
No	-	-	-	2.09	1.07–4.08	0.032
Clinical						
Psychiatric hospitalization						
Current	2.37	1.36–4.13	0.002	4.46	2.13–9.31	0.000
Prior	0.93	0.50–1.72	0.818	1.96	0.88–4.36	0.097
Never admitted	1.00			1.00		
Risk behavior						
Tobacco use (ever)						
Yes	5.57	2.64–11.75	0.000	2.47	1.12–5.42	0.025
No	1.00			1.00		
Alcohol use (ever)						
Yes	2.35	1.22–4.54	0.011	2.17	1.12–4.22	0.022
No	1.00			1.00		
History of homelessness						
Yes	1.75	1.17–2.63	0.007	-	-	-
No	1.00			-	-	-
Physical violence						
Yes	1.78	1.16–2.73	0.008	-	-	-
No	1.00			-	-	-
History of incarceration						
Yes	2.49	1.78–3.5	0.000	1.98	1.07–3.65	0.029
No	1.00			1.00		
Age of first sexual intercourse						
< 18 years old	1.66	1.07–2.58	0.023	2.39	1.23–4.63	0.010
≥ 18 years old	1.00			1.00		
Number of sexual partners						
One or never had sex	1.00			1.00		
Two or more	3.26	1.55–6.86	0.002	4.32	1.58–11.83	0.004

## DISCUSSION

Our study found higher rates of both past year (11.4%) and lifetime (25.4%) illicit drug use among psychiatric patients in relation to the rates in the Brazilian general population according to a household survey on drug use carried out in a similar period (2005) in collaboration with the Brazilian National Department on Drug Policies^1^. Moreover, we found higher proportions of specific drug use compared with the same study^1^ in the past year (8.8% and 2.6% for marijuana, 3.4% and 0.7% for cocaine, 2.4% and 1.2% for solvents, 5.4% and 0.1% for crack use, respectively) and lifetime (21.9% and 8.8% for marijuana, 10.6% and 2.9% for cocaine, 9.9% and 6.1% for solvents, 8.6% and 0.7% for crack use, respectively). Higher proportions of illicit drug consumption among psychiatric patients are also found in other countries when compared to the general population, with similar results^2,6,14,18,23^.. In a study carried out in London, Menezes et al.^18^ found that 43.3% of patients with SMI had used any drug during lifetime, and 4.7% of them presented some degree of substance use disorder in the year prior to the study. A review of the published literature on drug use among psychotic patients in the United Kingdom^2^ showed prevalence rates varying from 15% to 45% in the past year and from 16% to 68% for lifetime use.

In this study, for all substances, men had higher prevalence of use than women in the past year (marijuana: 14.2% *versus* 3.7%; cocaine: 5.6% *versus* 1.4%; solvents: 3.9% *versus* 0.9%; and crack: 8.8% *versus* 2.2%, respectively). Menezes et al.^18^ found similar results, in which men with SMI were 2.7 times more likely than women to have problems related to drug use. In Brazil, a household survey on drug use also indicated that men had a higher prevalence of use (lifetime) than women (marijuana: 14.3% *versus* 5.1%; cocaine: 5.4% *versus* 1.2%; solvents: 10.3% *versus* 3.3%, respectively)^1^.

We found that recent illicit drug use has strong and significant associations with alcohol and tobacco use for both men and women. Hasin et al.^10^ point out the consistency of associations between drug abuse, nicotine dependence, and alcohol use disorders even when controlling for sociodemographic characteristics and other comorbidities. De Leon et al.^15^ indicate that these overlapping disorders have important implications for treatment as patients with SMI who also abuse both alcohol and drugs rarely stop abusing just one of them.

The sociodemographic characteristics associated with drug use among patients with mental illnesses in our study are similar to those found in the general population. Our results are corroborated by previous studies that also indicate higher prevalence among men, younger persons^18^, and those with lower schooling^2,11^. Lower schooling was associated with recent use among men only, and professing a religion was negatively associated among women only. Professing a religion leads to the adoption of values and behavioral changes that restrict the use of illicit substances, thus acting as a protective factor^24^.

Studies have shown that patients with mental illnesses are sexually active and present high rates of sexual risk behavior^9,17,31^. In our study, we observed that recent illicit drug use was associated with younger age at sexual debut and having two or more sexual partners. The use of drugs may increase sexual risk behavior by modifying sexual impulses, thus increasing sexual desire, disinhibiting sexual behavior, or interfering with the practice of safer sex, or all of these^27^.

Illicit drug use was independently associated with a history of incarceration for both genders and with homelessness and physical violence for men. A British survey showed that 16% to 42% of the incarcerated population with psychosis presented dependence on some drug in the year prior to incarceration^4^. Persons with mental illness are more exposed to physical violence than the general population, which in turn is associated with illicit drug use, unstable housing or homelessness^21^, and a history of incarceration^16,21^. There is evidence that persons with mental illness are most often victims rather than perpetrators of violence^21^. However, some authors have suggested an increase in violent behaviors when illicit drug use is present^2^. These associations suggest the need to introduce new policies to address drug use and the need to prevent violence in mental health programs. We should note the existence of an important initiative in Brazil, named “doctor’s office in the street” (*Consultório de Rua*, in Portuguese), which provides mobile health units for the health care of the homeless and it attempts to link them to health care units for continuous follow-up^a^.

The association between current psychiatric hospitalization and illicit drug use in the past year found in our study is of clinical and management relevance. First, this finding corroborates the evidence that the association between mental disorders and drug use may lead to results of great severity^2^. Furthermore, according to the studies of Kessler^13^, a history of previous psychiatric hospitalization may indicate higher chances of developing future substance use disorders. The temporal relationship between the emergence of psychiatric and substance use disorders is widely discussed in the literature. Kessler et al.^14^ and Swendsen et al.^28^ indicate that the emergence of a psychiatric disorder typically precedes substance use disorder. However, in a literature review of studies with patients with schizophrenia, Gregg et al.^7^ reported no clear consensus regarding this matter.

This study is the first one to assess illicit drug use in a representative sample of psychiatric patients under care in public mental health services in Brazil. However, some limitations must be pointed out. The results presented here may not be generalized to all psychiatric patients because of the exclusion of more severely ill patients who were unable to participate. We did not directly assess psychiatric diagnoses or symptoms, but we rather obtained these data from medical charts. Patients who were treated exclusively at substance use centers (CAPS AD) were excluded, which might have led to the underestimation of the prevalence of illicit drug use among persons with mental illnesses. Despite the odds ratio being an appropriate measure for this study, it can potentially overestimate associations. In addition, the cross-sectional design of the study limits our capacity to establish a direct cause and effect and additional studies are necessary.

Our results have important implications for the psychiatric care in Brazil. The treatment of psychiatric patients who use illicit drugs is a major challenge for mental health services. Often professionals are not prepared to assist these persons, and services are not structured to provide high quality treatment or referral. There is a need for comprehensive integrated services to assist this vulnerable population. Strategies should be gender specific with a particular emphasis on the vulnerability to illicit drug use of men with mental illness.

## Footnotes

a Ministério da Saúde (BR). Consultório de Rua do SUS. Brasília (DF): Ministério da Saúde; EPIN-FIOCRUZ; 2010. Material de trabalho para a II Oficina Nacional de Consultórios de Rua do SUS; set 2010; Brasília (DF).
